# Comprehensive comparative genomics reveals over 50 phyla of free-living and pathogenic bacteria are associated with diverse members of the amoebozoa

**DOI:** 10.1038/s41598-021-87192-0

**Published:** 2021-04-13

**Authors:** Yonas I. Tekle, Janae M. Lyttle, Maya G. Blasingame, Fang Wang

**Affiliations:** Department of Biology, Spelman College, 350 Spelman Lane Southwest, Atlanta, GA 30314 USA

**Keywords:** Biodiversity, Coevolution, Bacterial infection

## Abstract

The Amoebozoa, a group containing predominantly amoeboid unicellular protists has been shown to play an important ecological role in controlling environmental bacteria. Amoebozoans not only graze bacteria but also serve as a safe niche for bacterial replication and harbor endosymbiotic bacteria including dangerous human pathogens. Despite their importance, only a few lineages of Amoebozoa have been studied in this regard. In this research, we conducted a comprehensive genomic and transcriptomic study with expansive taxon sampling by including representatives from the three known clades of the Amoebozoa. We used culture independent whole culture and single cell genomics/transcriptomics to investigate the association of bacteria with diverse amoebozoans. Relative to current published evidence, we recovered the largest number of bacterial phyla (64) and human pathogen genera (51) associated with the Amoebozoa. Using single cell genomics/transcriptomics we were able to determine up to 24 potential endosymbiotic bacterial phyla, some potentially endosymbionts. This includes the majority of multi-drug resistant pathogens designated as major public health threats. Our study demonstrates amoebozoans are associated with many more phylogenetically diverse bacterial phyla than previously recognized. It also shows that all amoebozoans are capable of harboring far more dangerous human pathogens than presently documented, making them of primal public health concern.

## Introduction

The study of microbial interactions is a complex and fascinating field of research^1–3^. Microorganisms occupy diverse ecological niches and are usually found in large communities that result in inherent interactions. Coevolutionary processes have been shaping these interactions, which gave rise to various types of adaptation, specialization and establishment of temporary and stable (obligate) associations^2,4–6^. Understanding microbial interactions have profound evolutionary implications; among other notable insights, it has contributed to our understanding of the origin of eukaryotic cells^7^, ecosystem health and function^8^ as well as disease and pathogen evolution^9–11^. While the biodiversity of microbes is generally poorly understood, many examples of well-established associations are known among various microbes^12^. Among these, the interactions of bacteria with protists (single-cell eukaryotes) have been a subject of immense scientific interest and substantial investigations^9–11,13,14^. Protists comprise some of the most important primary grazers of environmental bacteria. They play an integral role in major biogeochemical and ecological processes of microbial food webs, substantially contributing to nutrient recycling and energy transfer to higher trophic levels both in aquatic and terrestrial ecosystems^2,15^. Furthermore, many animal and human pathogenic bacteria are directly or indirectly associated with protists. Several studies have shown that many bacteria, including some that are well-known multi-drug resistant (e.g. *Legionella*), are capable of evading digestion by protists^3,16–18^. These bacteria use protist hosts as safe haven to reproduce and as intermediate agents to infect their final hosts. Many examples of this type of relationship are known in ciliates^13^, flagellates^19^ and amoeboids^3,9,15^. In this study, we will focus on the association of bacteria with the predominantly amoeboid supergroup, Amoebozoa.

The association of bacteria with Amoebozoa has been mostly studied from two representatives, *Acanthamoeba* and *Dictyostelium*^20–24^. These two amoebozoans are extensively studied as models in many important cellular processes and pathogenesis^10,21,25–28^. A substantial work on *Vermamoeba vermiformis* and sporadic reports on other amoebae (e.g. *Vannella, Arcella*) on association with bacteria are also available^18,21,29–33^. These studies demonstrated that amoebozoans are both grazers and hosts of some bacterial epibionts (attached to the surface of the amoebozoan) and endosymbionts (within the cytoplasm of the amoebozoan), the latter including dangerous human pathogens. Amoebozoans have been implicated as training ground for emerging pathogens and vehicles for their dispersal^4,21^. These studies also gave insights on mechanism of pathogen evasion and host defense^17,21,27,34^. Despite these major advances in the field, the number of amoebozoans examined for association with bacteria remain limited; and the studied lineages are not representative of the extremely diverse groups currently recognized within the supergroup. Amoebozoa encompasses members characterized by diverse morphology, ecology, behavior and life cycle^35–38^. The limited taxa used to study association with bacteria, undoubtedly has missed the vast diversity of bacteria that could potentially be associated with the Amoebozoa. Consequently, this under sampling hampers our knowledge of the positive contributions, and impact, that amoebozoans might have on the environment; and their role in major public health concerns.

Over ten amoeba-associated bacterial pathogens (in humans and other eukaryotes) belonging to the commonly discovered bacterial phyla (*Proteobacteria, Bacteroidetes, Chlamydiae, Firmicutes* and *Actinobacteria*) have been reported in the Amoebozoa^4,9,18,21,25,30^. Additionally, many non-pathogenic members of the above five phyla and some less known bacteria phyla (e.g. Candidatus *Dependentiae*), and unclassified or novel bacterial lineages, have been reported to form temporary or stable endosymbiotic associations with some amoebozoans^6,39,40^. These reports are mostly based on culture-dependent studies, which focus on the microbiome of bacteria that can be isolated and cultured independently or grown concurrently with the amoebozoan host. Culture-dependent studies fail to capture those bacteria that are unculturable under conventional laboratory conditions and with established culture media. Studies that used a culture-independent approach also suffer from limited taxon sampling, or they are limited to specialized or specific environments^30,41^. In order to capture the complete microbiome of the Amoebozoa-associated bacteria, we used a culture-independent, comprehensive genomic approach and surveyed 49 samples (38 species) covering most known lineages of Amoebozoa. The sampled organisms belong to the three major clades of Amoebozoa (Discosea, Evosea and Tubulinea), consisting of lineages of different morphology, ecology and behavior^35^. We used large molecular data, including genome data derived from whole culture and single cells maintained in our laboratory and transcriptome data obtained from prior published research. We assessed the impact of sampling and culturing conditions on the types and number of bacteria discovered. Our study reveals representatives from 64 bacterial phyla, including over 50 known human pathogenic species, potentially associated with the various members of the Amoebozoa. Our study reports the largest number of associated bacteria, including new phyla and pathogen genera, not reported in previous studies. Our findings reinforce previous reports that showed Amoebozoa as a major grazer of environmental bacteria, and host of many bacterial endosymbionts, some that pose a threat to public health. This study also lays foundation for further investigations on mechanisms of predator–prey relationships, evasion of host defense (immunity) and forms and types of associations of newly discovered epi- and endosymbionts, including internalized pathogenic bacteria.

## Results

### Overall composition of amoebozoa associated bacteria

**Figure 1:**
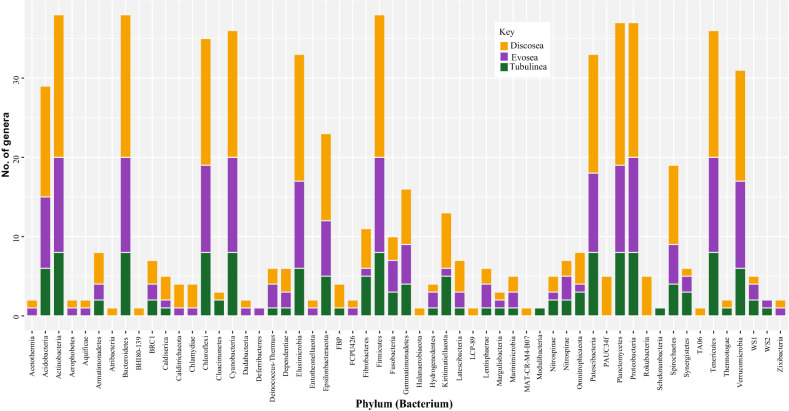
Distribution of sequences identified (number of genera) representing the 57 Bacterial phyla discovered in the three major clades of Amoebozoa across all datasets analyzed using Kraken 2.

**Figure 2:**
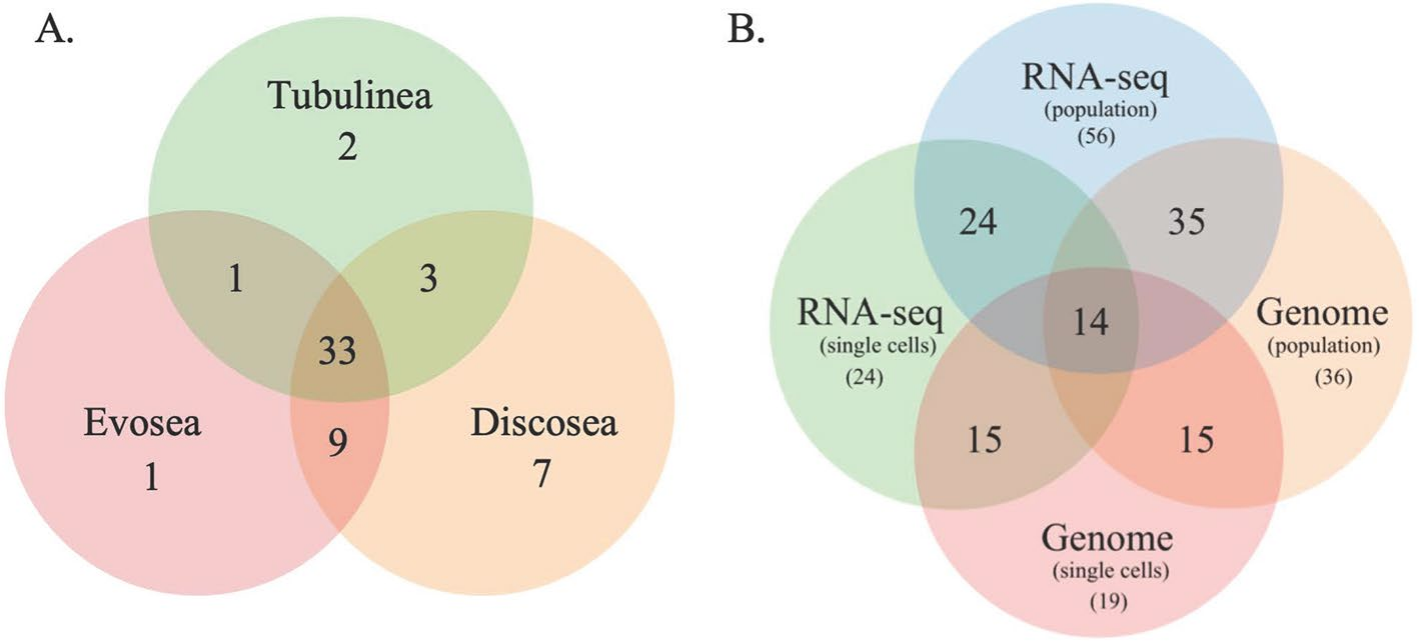
Venn diagram showing bacterial phyla shared among the three major clades of Amoebozoa of the whole culture RNA-Seq data (**A**) and among the four types of datasets analyzed (**B**) analyzed using Kraken 2.

**Figure 3:**
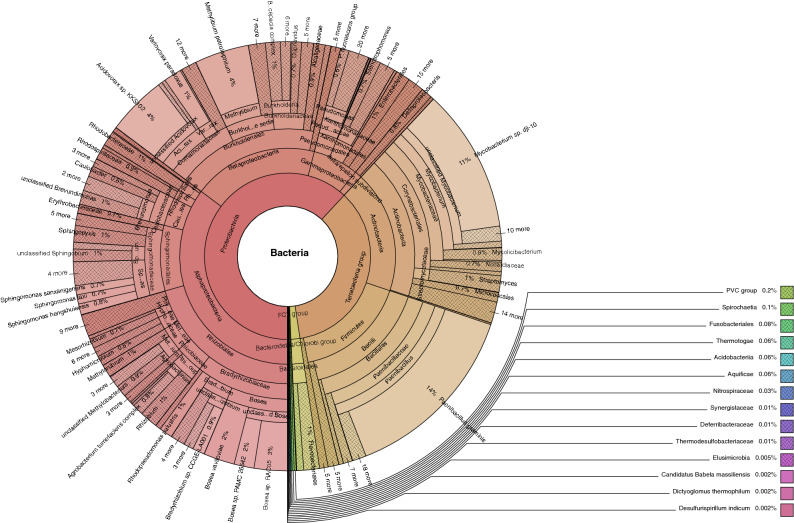
Krona plot of bacterial sequences identified in the whole culture genome data (10× genomics) of *Cochliopodium minus* analyzed using Centrifuge.

Taxonomic assignment of the various genetic datasets analyzed, combining genome data generated in this study with transcriptomes from previous studies, yielded a large number of amoebozoan-associated representative bacteria phyla with overall similar taxonomic compositions across the three clades of Amoebozoa and methods used (Tables S1-S3). Both Kraken 2 and Centrifuge analyses recovered a similar number of bacteria phyla across samples analyzed (Tables S1-S3). Overall Centrifuge yielded more bacterial phyla than Kraken 2 except for few samples (Fig. S1, Tables S1-S4). This is expected since the data used in Centrifuge included non-ribosomal genomic and transcriptomic data. The classification and number of bacterial phyla covered in Kraken 2 and Centrifuge databases are slightly different (Fig. 3, Table S1). The results are reported from both analyses side by side in our supplementary Table S1 using common taxonomic naming. Since the results from these methods are similar and 16S ribosomal gene is commonly used for species identification in bacteria, we will report results mostly based on Kraken 2 analysis. A combined total of 61 bacterial phyla (57 in Kraken 2 analysis) were discovered from all of the datasets examined (Figs. 1, 2, 3, Tables S1-S3). Since the majority of bacterial phyla, 56, were found in the whole culture RNA-Seq dataset, we will focus our comparison among the clades of Amoebozoa based on this dataset mostly (Fig. 2). One additional phylum, *MAT-CR-M4-B07*, besides others was found in the whole culture genome dataset (Table S3). Discosea, with the highest number of taxa analyzed in whole culture RNA-seq dataset, had 52 (60 in Centrifuge) associated bacterial phyla, while Evosea and Tubulinea had 44 (53 in Centrifuge) and 39 (49 in Centrifuge) phyla, respectively (Fig. 1, Table S1). Among these discovered phyla, 33 phyla are shared among the three clades (Fig. 2A). While the bacterial taxon sampling for Tubulinea in the transcriptome data is smaller than Evosea and Discosea, the latter two clades shared more bacterial phyla between them (i.e. 9), when compared to the phyla that they each mutually shared with Tubulinea (i.e. 1 and 3, respectively) as shown in Fig. 2A. We also found some bacterial phyla specifically associated with each clade; namely, 7 in Discosea, 2 in Tubulinea and 1 in Evosea (Fig. 2A, Table S1). However, in future research, the specific representative bacterial phyla recovered in each clade might change with more taxon sampling, and in relation to the nature of the acquired data. For instance, two samples from the same species, *C. minus*, in the whole culture genome data showed variation in the number of bacterial phyla recovered and shared (Table S3). This indicates that a thorough and even sampling is required to make such comparisons. Overall, phyla recovered were proportional to data size and taxon sampling (Fig. 1, Tables S1-S3).

The total number of genera and their representation differed by bacterial phyla in our datasets. The most abundant bacterial phylum recovered in all datasets and amoebozoan clades is *Proteobacteria* (Tables S1-S3). Class *Gammaproteobacteria*, a subdivision of *Proteobacteria*, was represented by a higher number of genera and total number of sequences that were representative for its genera (Tables 1, S1-S4). Other bacterial phyla that were represented by over 1000 sequences for the genera recovered, in Kraken 2 analysis, include *Bacteroidetes* and *Firmicutes* (Table S1). Generally, a higher number of sequences representing a given phylum were observed in the whole culture genome data (Fig. 3, Table S3).

**Table 1:** List of potential human pathogens discovered in all analyzed samples. Numbers of amoebae samples possessing listed pathogens are shown in parenthesis.

Phylum	Genus	Species
Actinobacteria	*Corynebacterium*	*C. diphtheriae* (18)*, C. jeikeium* (20)*, C. urealyticum* (11)
	*Gardnerella*	*G. vaginalis* (26)
	*Mycobacterium*	*M. leprae* (11)*, M. tuberculosis* (16)
	*Nocardia*	*N.brasiliensis* (23)
Bacteroidetes	*Bacteroides*	*Bacteroides fragilis* (30)
	*Capnocytophaga*	*C. canimorsus* (26), *C. ochracea* (17)
	*Porphyromonas*	*P. gingivalis* (17)
Chlamydiae	*Chlamydia*	*C. pneumoniae* (11)*, C. trachomatis* (12), *C. psittaci* (7)
Firmicutes	*Bacillus*	*B. anthracis* (30)*, B. cereus* (43)*, B. subtilis* (43)
	*Clostridium*	*C. botulinum* (43)*, C. perfringens* (38)*, C. tetani* (30)
	*Enterococcus*	*E. faecalis* (30)*, E. faecium* (31)
	*Listeria*	*L. monocytogenes* (35)
	*Staphylococcus*	*S. aureus* (39)*, S. epidermis* (36)*, S. hemolyticus* (29)*, S. saprophyticus* (26)
	*Streptococcus*	*S. pneumoniae* (33), *S. agalactiae* (30)*, S. pyogenes* (30), *S. viridans* (2)
Fusobacteria	*Fusobacterium*	*Fusobacterium nucleatum* (45)
**Proteobacteria**		
Alpha-	*Anaplasma*	*A. phagocytophilum* (9)
	*Bartonella*	*B. bacilliformis* (13)*, B. henselae* (15)*, B. quintana* (12)
	*Brucella*	*B. suis* (15)
	*Ehrlichia*	*E. canis* (21)*, E. chaffensis* (20)
	*Rickettsia*	*R. rickettsii* (4)
Beta-	*Achromobacter*	*A. xylosoxidans* (36)
	*Bordetella*	*B. pertussis (22)*
	*Burkholderia*	*B. cepacia* (35)*, B. pseudomallei* (39)
	*Neisseria*	*N. gonorrhoeae* (14)*, N. meningitidis* (35)
Epsilon-	*Campylobacter*	*C. fetus* (28)*, Campylobacter jejuni* (36)
	*Helicobacter*	*H. pylori (42)*
Gamma-	*Acinetobacter*	*A. baumannii* (40)
	*Aeromonoas*	*A. hydrophila* (40)*, A. veronii* (21)*, A. schubertii* (20)
	*Citrobacter*	*C. koseri* (16)*, C. freundii* (34)
	*Coxiella*	*C. burnetii* (30)
	*Enterobacter*	*E. cloacae* (35)
	*Escherichia*	*E. coli* (44)
	*Francisella*	*F. tularensis* (32)
	*Haemophilus*	*H. influenzae* (36)
	*Klebsiella*	*K. pneumoniae* (41)
	*Legionella*	*L. pneumophila* (38)
	*Moraxella*	*M. catarrhalis* (23)
	*Morganella*	*M. morganii* (16)
	*Pasteurella*	*P. multocida* (24)
	*Proteus*	*P. mirabilis* (24)*, P. vulgaris* (19)
	*Providencia*	*P. stuartii* (23)
	*Pseudomonas*	*P. aeruginosa* (40)
	*Salmonella*	*S. enterica* (39)
	*Serratia*	*S. marcescens* (40)
	*Shigella*	*S. dysenteriae* (16)*, S. flexneri* (34), *S. sonnei* (31)
	*Vibrio*	*V. cholerae* (30)*, V. parahaemolyticus* (38)*, V. vulnificus* (33)
	*Yersinia*	*Y. pestis* (28)
Spirochaetaes	*Borrelia*	*B. recurrentis* (9)
	*Leptospira*	*L. borgpetersenii* (31)*, L. interrogans* (34)*, L. santarosai* (20)
Tenericutes	*Treponema*	*T. pallidum* (15)
	*Mycoplasma*	*M. pneumoniae* (14)

Species level identification of pathogens was based on Centrifuge analysis; the true nature of association and identification should be confirmed further by experimental evidence.

### Comparison of data types and potential endosymbiont bacterial phyla

The four data types, genome (whole-culture and single cells) and RNA-Seq (whole-culture and single cells), analyzed yielded bacterial phyla that are commonly shared among samples and amoebozoan clades analyzed (Figs. 2, S1, Tables S1-S3). We observed some variations in taxonomic breadth and the total number of genera/species recovered depending on data type and taxon sampling size (Figs. 2, S1, Tables S1-S3). As mentioned above all except one bacterial phylum reported here were present in whole culture RNA-Seq datasets (Table S1). While the large number of bacterial phyla in the whole culture RNA-Seq dataset can be partly attributed to the size of taxon sampling used for this dataset, these results clearly indicate that RNA-Seq is a good data source for this type of study. The whole culture genome data is represented by two independent samples from a single species, *C. minus* (Table S3). A total of 36 bacterial phyla were recovered from these two samples, 35 of these are shared with the whole culture RNA-Seq dataset (Fig. 2B). The single cell genome data yielded the lowest number,19 bacterial phyla (Table S3), after the single cell RNA-Seq data (24 phyla) (Table S2). Using the four datasets we were able to identify 14 potential endosymbionts/epibionts by taking a subset of the bacterial phyla discovered in each dataset (Fig. 2B). Use of single cells datasets, both genome and RNA-Seq, primarily aimed at reducing bacteria contamination from external environment, enabled us to deduce these 14 putative endosymbionts/epibionts. A total of 24 potential endosymbionts/epibionts phyla can be recognized if we considered taxa shared among three datasets i.e. all the phyla discovered in single cell RNA-Seq dataset (Fig. 2B). Among these seven putative endosymbiont phyla (5 shared in all and 2 shared among 3 datasets, see also Tables S1-S3) included members (human pathogen genera/species) previously reported to associate with or found in the cytoplasm of amoebozoan hosts^3,9,23,25,34,42^. Centrifuge analysis yielded the same or less number of bacterial phyla from the genome data compared to the Kraken 2 analysis (see Tables S1-S3, Fig. S1). The numbers of bacterial phyla recovered in the RNA-Seq datasets were comparable between the two approaches, with Centrifuge recovering more phyla than Kraken 2 (Tables S1, S2).

In order to assess the impact of culturing techniques and types of bacteria that may be associated due to difference in the environment of isolation and types of food sources used between labs, we compared RNA-Seq data of three taxa sequenced in two different labs. Our comparison showed a similar total number of bacterial phyla recovery but with some differences in the number of overlapping phyla (Table S1). The variation of non-overlapping phyla in these three pairs of species ranged from 5 to 7. This observed difference using the RNA-Seq data is smaller compared to the variation observed in the number of non-overlapping phyla found in the genome data samples (Table S3). The whole culture genome data used two samples from the same species that were cultured under the same conditions. These two samples had 9 non-overlapping bacterial phyla, which indicate that other technical factors, such as sample quality (e.g. starting RNA/DNA material) and sequencing (e.g. depth/coverage), might affect the recovery rate of overlapping bacterial community in samples of the same species.

### Human pathogenic bacterial phyla and genera associated with amoebozoa

Our survey of literature and databases (e.g., https://globalrph.com/bacteria/) for bacterial pathogens yielded over 50 known and potential human pathogen genera along their representative species (Table S4). We used this list to investigate the presence of pathogenic species in our datasets (Tables S4). We used both Kraken 2 and Centrifuge to analyze our samples for pathogens, but Kraken 2 taxonomic classifications is only to a genus level and will not be discussed further. Of the human pathogenic genera surveyed, over 50 genera spanning eight different bacterial phyla were recovered (Fig. S2, Table 1, Table S4). The number of pathogens recovered in the three clades was the same (51 pathogenic genera) in all clades despite taxon sampling differences in the whole culture RNA-Seq dataset (Table S4). These genera are represented by 84 well-known and described species in Discosea and Evosea, and 85 species in Tubulinea (Table S4). One extra species, *Streptococcus viridans*, was found in one species of Tubulinea (*Cryptodifflugia operculata,* Table S4). The detection of pathogens across clades and samples was uniform in the RNA-Seq dataset (Table S4). The minimum number of pathogen genera in this dataset was 23, which was recorded in two members of Discosea (*Parvamoeba monoura* (YT) and *Stenamoeba limacina*). Interestingly, the number of pathogens recovered from another, *Parvamoeba monoura,* sequenced in a different lab (Kang et al.^35^) had much higher associated pathogens (38 genera) than the one sequenced in our lab. Similar number of pathogenic genera (26–49) were recovered from whole culture genome and single cell transcriptome datasets (Table S4). The single cells genome data yielded the least pathogenic genera (0–8), which is similar to results found in Kraken 2 analysis (data not shown).

The top three phyla with the highest number of pathogenic bacterial genera (4 or more genera and associated species per phylum) recovered include *Proteobacteria*, *Actinobacteria* and *Firmicutes* (Table 1). Among the classes of *Proteobacteria*, *Gammaproteobacteria* had the largest number (21 pathogen genera, 29 species) compared to any group analyzed (Table 1). Majority of the detected pathogen genera are found in several amoebae belonging to the three major clades of Amoebozoa (Table 1, S4, Fig. S2).

## Discussion

### Large amoebozoa associated bacterial phyla recovered

Our study using whole culture and single cell genomics and transcriptomics recovered the largest number of bacterial phyla that are potentially associated with the supergroup Amoebozoa to date. The majority of the known bacterial phyla (~ 50) recovered in our analysis of the amoebozoans are reported here for the first time (Fig. 1, Tables S1-S3). We also found well known and common amoebozoan-associated bacterial phyla (5–10) reported in previous studies^3,4,6,9,16,25,29–31,41^. The large and taxonomically diverse discovery of amoebozoans associated bacterial phyla in this study could be attributed to the comprehensive taxon sampling and molecular genetic approach employed. We analyzed amoebozoans characterized by diverse ecology, behavior and evolutionary history that represented the three major clades of the Amoebozoa. We used monoclonal cultures of amoebozoans isolated directly from nature or acquired from culture collection agents^35,36,38^. Research methods using monoclonal cultures typically include addition of food bacteria (e.g., *E. coli* or *Klebsiella*); but once the culture starts to advance, it is common to see more bacterial communities, besides food bacteria, growing among the amoebozoan cells. Amoebozoans are known to carry undigested food bacteria vertically through generations^43^. These food bacteria are used presumably as seeds to be conserved for potential replenishment within new environments encountered by the amoebozoan, and then harvested as food; this behavior led some to metaphorically call amoebozoans, ‘farmers’^21,43–45^. Therefore, the bacteria found in monoclonal samples analyzed likely reflect a bacterial community that might be expected to occur naturally in nature; although we cannot rule out that some are acquired from contamination during laboratory culture as for example from contact with instruments used in culturing or from air-borne bacteria introduced from the laboratory environment. The taxonomic composition of bacteria found in amoebozoans grown in different labs, or obtained from different culture collection agents or nature, in the RNA-Seq data were similar (Table S1). The consistent recovery of similar bacterial phyla across different amoebozoan samples and taxonomic groups, that we have found in our analyses for this research study, also indicates that all bacterial lineages discovered in our analysis are potentially associated with the Amoebozoa, and may mitigate against possible contamination from sources largely derived from the laboratory environment. While the confirmation and type of association of the newly discovered bacteria awaits further investigation, our study reinforces amoebozoans as key players in controlling environmental bacteria through grazing. Our study also suggests that Amoebozoa potentially can harbor more taxonomically diverse bacteria, with 64% of the 89 bacterial phyla in SILVA database recovered, than previously reported.

The large taxonomic sampling of amoebozoans in our study was made possible by the use of transcriptome data. In recent phylogenomic studies, a large number of RNA-Seq datasets have been generated in the Amoebozoa^35,36,38^. These transcriptome data are generated using a standard approach that selects polyadenylated RNA (polyA) in RNA samples, which selects against bacterial contaminant transcripts that are typically poorly polyadenylated^46,47^. However, transcriptome data collected from amoebozoans using this approach typically contains large bacterial transcripts and some ribosomal genes^35,36,38^. While contamination by bacteria in transcriptome data has been reported in axenic culture, or in species that do not normally feed or associate with bacteria (likely contamination from environment)^48^, the close association of bacteria (food and endosymbiont) with amoebozoans exacerbates the potential for contamination of transcriptomes even more. We took advantage of this, and used the 16S bacterial ribosomal genes found in amoebozoan RNA-Seq data to assess bacterial association with the Amoebozoa. Despite the potential limitation that transcriptome data might have for our study, the aggregate number of bacterial phyla recovered from transcriptome sequencing was comparable in taxonomic coverage to the whole culture genome data (Fig. 2). As expected, the number of genomic representations of the discovered phyla in the whole culture genome data was higher than the transcriptome data (Tables S1-S3), which indicates that transcriptome data might to an extent underrepresent the actual diversity of associated bacterial populations. But when bacterial transcripts were analyzed along the 16S ribosomal genes in our Centrifuge analysis, the number of bacterial phyla recovered increased by greater than eight (8–11) for all clades examined (see Table S1). Our results support the utility of transcriptome data to study association of bacteria with amoebozoans or other similar protists. Though a conservative estimate, transcriptome data has some advantages over genome data due to lower cost and ease in acquiring it. Moreover, transcriptome data can provide additional information on the nature of an association by providing physiological data (profile of expressed genes) among interacting species^49^.

In addition to the rich sources of transcriptome data as discussed above, the use of whole culture and single cell genomics, as used in our laboratory culture studies reported here, enabled us to assess potential bacterial endosymbionts (possibly including epibionts) associated with the Amoebozoa. Using this approach, we identified 14–24 potential endosymbionts/epibionts bacterial phyla (Fig. 2B, Tables S1-S3). Our list includes bacteria phyla whose members were previously shown to form true endosymbiotic relationships in some amoebozoans^6,9,29,50,51^. However, a more thorough approach including single cell genome and cytological data, such as use of fluorescently labeled oligonucleotide probes^29^, is needed to establish true endosymbiotic relationships with Amoebozoa. Nonetheless, the recovery of known endosymbiotic bacteria in our analysis gives credence to the reliability of our approach to identify potential endosymbiotic bacteria candidates that can be studied further. It should be noted that some amoebozoans are selective bacterial predators^52–54^. The combination of single cell genomics and transcriptomics approaches used here is a promising method of analyzing selective feeding on bacteria by protists; e.g., a recent study demonstrated the utility of transcriptome data for selective feeding in a ciliate lineage^49^.

### Pathogenic bacteria associated with the Amoebozoa

The association of pathogenic bacteria with some members of Amoebozoa has been investigated in great detail^3,4,21,22,27,55^. Most of the association of pathogenic bacteria described with amoebozoans is facultative, but some permanent associations are also known^6,29,42^. While most associations are transient and harmless, some bacterial infections (e.g. *Legionella*), leading to lysis of amoebozoan cells, have been reported^4,56^. In facultative associations, the pathogenic bacteria can use the amoeba cell as a safe niche to reproduce, or intermediate host, or even as a vehicle for dispersal or population reservoir^4,22^. Some recent studies have proposed that amoebozoans could serve as an ‘interim training ground’ to develop intracellular survival strategies before becoming a human pathogen due to the similarity in mechanism of phagocytosis (phagolysosome) within mammalian macrophages^4,17,28^. Most of the known pathogenic bacteria associated with Amoebozoa so far come from the studies that used only a few amoebozoan species, which are not necessarily reflective of pathogens that can potentially be harbored by various groups in the supergroup of Amoebozoa. In this study, we discovered 51 pathogenic bacterial genera (85 species) belonging to eight phyla, the highest report so far (Table 1). The number and distribution of pathogenic genera across the three major groups of Amoebozoa were comparable despite differences in taxon sampling among them (Figs. S1, S2). Our list includes previously reported common representatives of pathogen bacterial phyla^21,55^ in addition to the large number of pathogens newly discovered in this study (Tables 1, S4). Congruent with previous studies, the most dominant pathogen-containing phylum is *Proteobacteria*. One of its subdivisions, class *Gammaproteobacteria*, comprised more than 40% of the pathogenic genera identified in this study (Table 1). The representation of some pathogen-containing phyla might be affected by habitat examined. Nevertheless, our results demonstrate that all amoebae are potential carriers of bacterial pathogens both in nature or anthropogenic environments. All of the multi-drug resistant species found in this study are listed and categorized by CDC and WHO as urgent, and various levels of threats and concerns. Among these are *Acinetobacter baumannii, Enterococcus faecalis, Clostridium spp., Neisseria gonorrhoeae, Helicobacter pylori, Campylobacter spp., Pseudomonas aeruginosa, Salmonella enterica, Mycoplasma pneumoniae, Staphylococcus aureus, Haemophilus influenzae, Streptococcus pneumoniae* and *Bordetella pertussis,* which were found in most of the amoebozoan samples we examined (see Table 1). This makes some Amoebozoa that are associated with potential or acknowledged human pathogens a major public health threat.

## Materials and methods

### Amoebae cultures

Amoebae cultures used for genomic data in this study come from different sources including ATCC (*Cochliopodium minus* ATCC 30935,* Trichosphaerium* sp. ATCC 40318), Ward’s Science culture collection (wardsci.com, *Amoeba proteus*) and a newly described species isolated from mixed eukaryotic culture in our lab (*Stratorugosa tubuloviscum*). All these cultures have been maintained in our lab. *Stratorugosa tubuloviscum* and *C. minus* were grown in plastic Petri dishes with bottled natural spring fresh water (Deer Park, Nestlé Corp. Glendale, CA, USA) with added autoclaved grains of rice as an organic nutrient source to support bacterial growth as prey for the amoebozoans. The marine amoeba, *Trichosphaerium* sp., was grown under a similar condition as above in artificial seawater prepared by mixing 1 ml of distilled water in 30 g of Instant Ocean (Cincinnati, OH) sea salt. *Amoeba proteus* was cultured with mixed bacteria and other microbial eukaryote food sources.

### Whole culture and single cell genomics

We used various approaches to investigate bacteria associated with amoebozoans. Association of bacteria with their host can be internal endosymbionts or external those that are epibionts attached to the surface of the cell and those that are freely present in cultures that are potentially available to be engulfed as a food source. In order to capture all associated bacteria in diverse monoclonal cultures of amoebozoans in our laboratory, we used molecular data collected using two approaches. The first set of genetic data collected consisted of community genomic DNA extracted from actively growing cultures of amoebozoans; and from the bacterial community typically found in monoclonal or newly isolated species maintained in our laboratory cultures. The second genetic data is derived from single amoebozoan cells, individually picked out of our laboratory cultures. The main difference between these two approaches is that the first approach, *whole culture*, is aimed at collecting large quantities of DNA from a monoclonal population without little consideration to bacteria contamination from the culture; while the second approach, *single cell*, is aimed at minimizing bacterial contamination from the surrounding environment/culture.

In the single cell approach amoebozoan cells, which include *Cochliopodium minus, Stratorugosa tubuloviscum, Trichosphaerium* sp. and *Amoeba proteus* were individually picked using mouth pipetting techniques and transferred to a clean glass slide to wash off bacteria (other microbial eukaryotes (food or prey) in *A. proteus* culture) to reduce contamination of freely growing bacteria (other contaminants) from the culture. This step does not necessarily remove epibionts that are tightly bound to the cell surface but it greatly minimizes free (loosely bound) bacteria growing in culture. Cleaned individual cells (5–10) were transferred into 0.2-mL PCR tubes and genome amplified using REPLI-g Advanced DNA Single Cell Kit (Qiagen Hilden, Germany).

For the whole culture approach, genomic DNA was extracted from a large number of *Cochliopodium minus* (syn. *C. pentatrifurcatum*^57,58^ cells in culture dishes (50 Petri dish cultures) using MagAttract high-molecular-weight (HMW) DNA kit (Qiagen, MD), following the manufacturer’s instructions. This method includes gentle cell lysis, releasing high molecular weight DNA and its efficient isolation and purification by concentration on DNA-binding, surface coated magnetic beads. Genome sequencing was performed using 10X genomics (for whole culture DNA) and Oxford Nanopore (ONP) (for both single cells and whole culture DNA) following the manufacturers’ protocol. Genome data from 10X genomics and ONP were assembled using Supernova v2.1.1^59^ and Minimap2-Miniasm-Racon genome assembly pipeline^60–62^, respectively. For ONP genome data we used Porechop version 0.2.4 (https:// github.com/rrwick/Porechop) to remove ONP sequencing adapters added during the sequencing.

Filtlong version 0.2.0 (https://github.com/rrwick/Filtlong) was used to filter reads with length shorter than 200 and quality score less than 5.

### Whole culture and single cell transcriptome data

Based on preliminary analysis that showed amoebozoan transcriptomes contained large bacterial transcripts and some ribosomal genes, we analyzed RNA-Seq from previous publications that were collected in a similar manner as above^35,36,38,63^. The samples from previous publications included amoebozoans that were grown in established laboratory cultures and single cells isolated from these culture as well as single cells directly isolated from various environments (see Table S5)^35,36,38,63^.The whole culture RNA-Seq dataset included a total of 35 species (15 discoseans, 12 evoseans, and 8 tubulinids) with three additional duplicate samples from Discosea sequenced in two different labs^35,36,38^. These discosean duplicate samples were included in the analysis to examine the effects of culturing methods and environment on the number and composition of bacterial community recovered. The single cell RNA-Seq dataset was represented by five samples obtained from *Cochliopodium minus*^64^. Data collection, sequencing and assembly of transcriptome data of these diverse amoebozoans, representing the three main clades of Amoebozoa (Discosea, Evosea, and Tubulinea) of the whole culture and single cell RNA-Seq datasets, are described in Kang et al.^35^ and Tekle et al.^36,38^, and Tekle et al.^63^, respectively. Some good quality transcriptomes whose origin was not certain or is collected using a combination of single cell and whole culture are placed in the whole culture RNA-Seq dataset (Table S5). All transcriptomes used for single cell RNA-Seq dataset including five replicate samples from *C. minus* (Table S3) are collected in our laboratory under similar experimental conditions^64^.

### Taxonomic assignment of amoebozoa associated bacterial sequence data

We used two taxonomic assignment tools, Kraken 2^65^ and Centrifuge^66^, commonly used in metagenomic studies. A total of 49 samples (genome and transcriptome data) of amoebozoans, representing 38 species belonging to the three major clades of Amoebozoa were analyzed. Similarly, we compared taxonomic composition in four datasets, which include two genome (whole-culture and single cells) and two RNA-Seq (whole-culture and single cells) data types. Kraken 2 with default settings, shown to have high sensitive and accuracy^65^, was used to analyze the assembled contigs from genome and transcriptome data. Kraken 2 classifies sequences by mapping k-mer to the lowest common ancestor (LCA) of all the datasets containing the given k-mer in the specified database. The 16S database, SILVA, was chosen for this analysis and taxonomic classification was done to a genus level. Kraken 2 was run locally in an interactive session on XSEDE server, a supercomputing platform (http://xsede.org). We also conducted similar analysis using Centrifuge, a rapid and accurate metagenomics classifier that uses the Burrows–Wheeler transform (BWT) and an FM-index to store and index the genome database^66^. We used pre-built database index from Centrifuge website constructed from complete Bacteria, Archaea, Viruses and Human genomes from NCBI GenBank (as of 2016). Centrifuge allowed us to use raw (not reported) and assembled non-ribosomal genomic and transcriptomic data in addition to the ribosomal (16S) used in the Kraken 2. Centrifuge also can identify sequences to species level when sufficient matches are found. Results from Centrifuge analysis was visualize using Krona^67^, an online interactive metagenomic visualization program. Resulting data were further analyzed using R and Excel. Data used in this study are available upon request.

## Supplementary Information

Supplementary Legends

Supplementary Figure 1.

Supplementary Figure 2.

Supplementary Table 1.

Supplementary Table 2.

Supplementary Table 3.

Supplementary Table 4.

Supplementary Table 5.
