# Supplementary material for: Comprehensive comparative genomics reveals over 50 phyla of free-living and pathogenic bacteria are associated with diverse members of the amoebozoa
Source: Sci Rep. 2021 Apr 13;11:8043. doi: 10.1038/s41598-021-87192-0 (PMC8044228; doi:10.1038/s41598-021-87192-0)
Supplement: Supplementary file 1 — Supplementary Legends [file 41598_2021_87192_MOESM1_ESM.docx]

**Supplementary Materials Captions**

**Figure S1**. Number of bacterial phyla representatives recovered from all examined amoebae taxa and data sources analyzed using Kraken 2 (blue bars) and Centrifuge (orange bars).

**Table S1**. Tally of genera representing each bacterial phylum in whole culture RNA-Seq dataset. The detection of bacterial genera are showing by total number of representatives in each phylum/sample in Kraken 2 and presence (+) or absence (-) in Centrifuge analyses. All amoebozoans representing the three major clades including species pairs sequenced in different labs (shown in red font) are included.

**Table S2**. Tally of bacterial genera derived from single cells RNA-Seq dataset. For this analysis five replicates samples from *Cochliopodium minus* were examined. The detection of bacterial genera are showing by total number of representatives in each phylum/sample in Kraken 2 and presence (+) or absence (-) in Centrifuge analyses.

**Table S3**. Tally of bacterial genera derived from whole culture and single cell genome datasets. The detection of bacterial genera are showing by total number of representatives in each phylum/sample in Kraken 2 and presence (+) or absence (-) in Centrifuge analyses.

**Table S4**. Tally of potential human pathogenic bacterial genera and species for all datasets analyzed using Centrifuge.

**Table S5**. Taxa studied and data sources.
