# Supplementary figures and images for: Comprehensive comparative genomics reveals over 50 phyla of free-living and pathogenic bacteria are associated with diverse members of the amoebozoa
Source: Sci Rep. 2021 Apr 13;11:8043. doi: 10.1038/s41598-021-87192-0 (PMC8044228; doi:10.1038/s41598-021-87192-0)

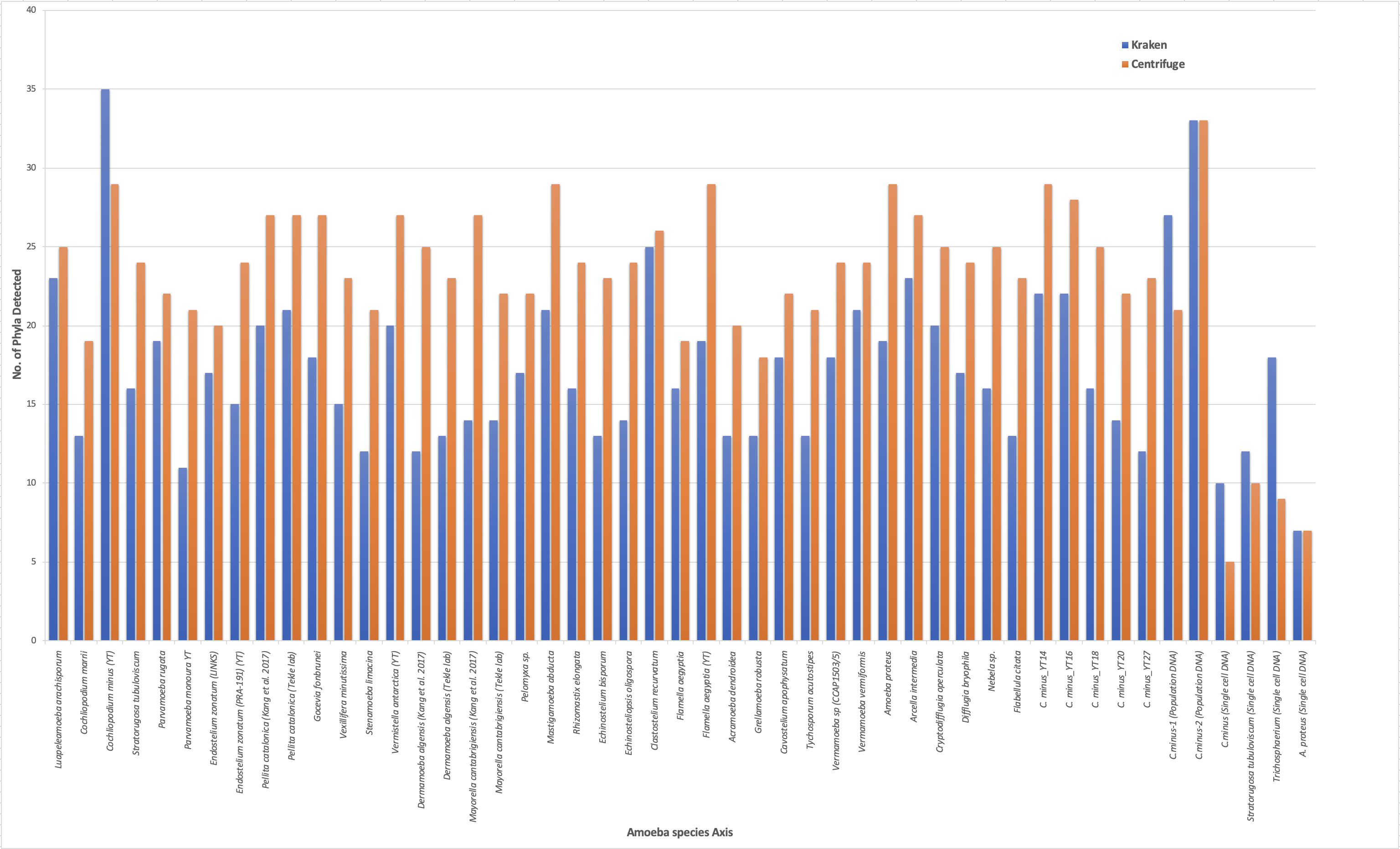

Supplement: Supplementary file 2 — Supplementary Figure 1. [file 41598_2021_87192_MOESM2_ESM.png]

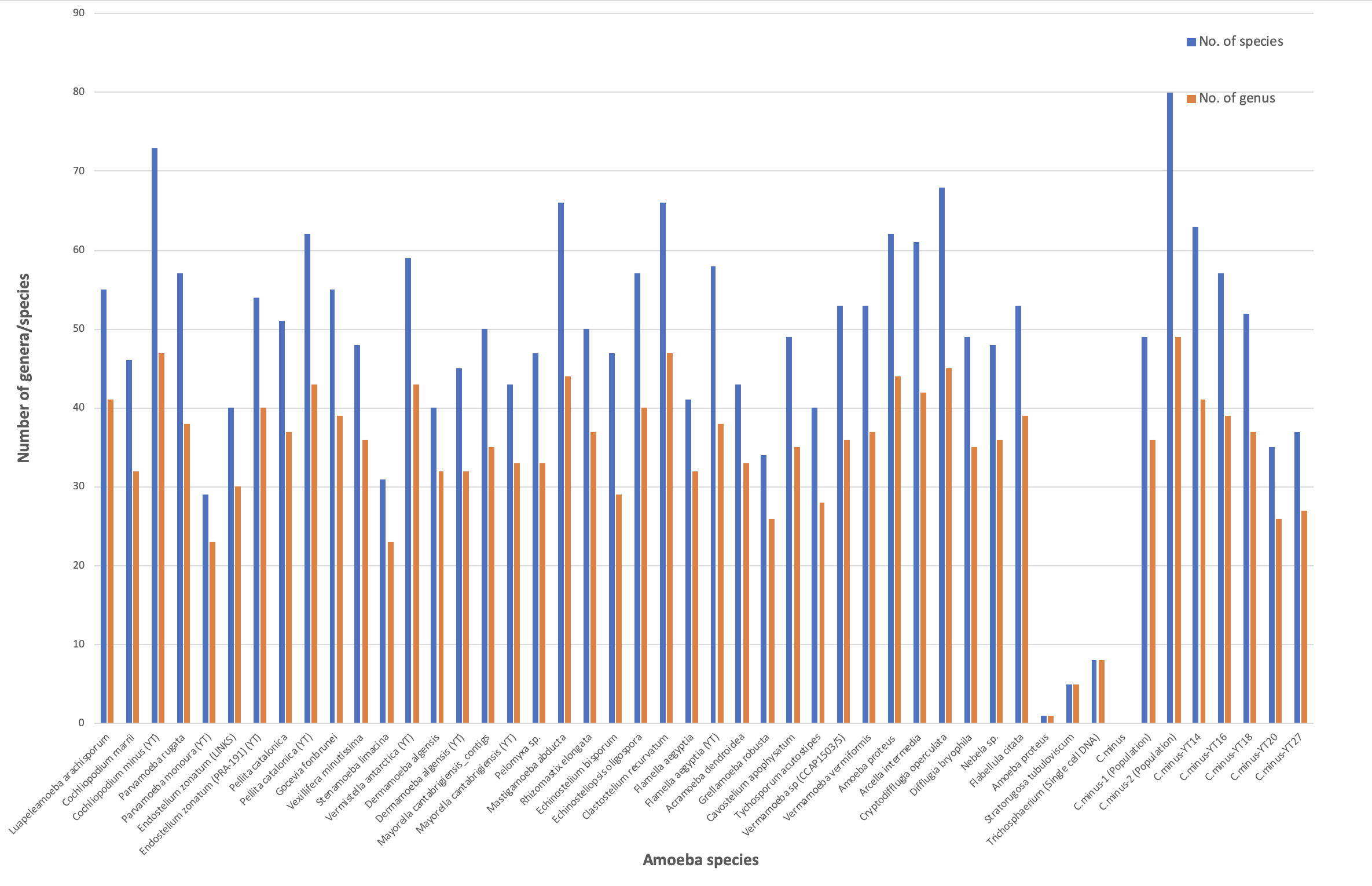

Supplement: Supplementary file 3 — Supplementary Figure 2. [file 41598_2021_87192_MOESM3_ESM.png]
